# *N*-[(4-Methyl­phen­yl)sulfon­yl]acetamide

**DOI:** 10.1107/S1600536812024658

**Published:** 2012-06-13

**Authors:** Hoong-Kun Fun, Tze Shyang Chia, Poornima Hegde, K. Jyothi, Pramila Rita D’Souza

**Affiliations:** a X-ray Crystallography Unit, School of Physics, Universiti Sains Malaysia, 11800 USM, Penang, Malaysia; b Department of Chemistry, St Joseph Engineering College, Vamanjoor, Mangalore 575 028, Karnataka, India

## Abstract

In the title compound, C_9_H_11_NO_3_S, the dihedral angle between the benzene ring and the amide group is 76.7 (3)°. In the crystal, mol­ecules are linked by pairs of C—H⋯O hydrogen bonds into inversion dimers with *R*_2_^2^(8) ring motifs. The dimers are further connected by N—H⋯O and C—H⋯O hydrogen bonds into an infinite tape running parallel to the *b*-axis direction.

## Related literature

For details of the biological activity of sulfonamides, see: Kamoshita *et al.* (1987 ▶); Heidler & Link (2005 ▶); Ashton *et al.* (1994 ▶). For related structures, see: Henschel *et al.* (1996 ▶); Gowda *et al.* (2007 ▶, 2010 ▶); Shakuntala *et al.* (2011*a* ▶,*b* ▶). For the stability of the temperature controller used in the data collection, see: Cosier & Glazer (1986 ▶).
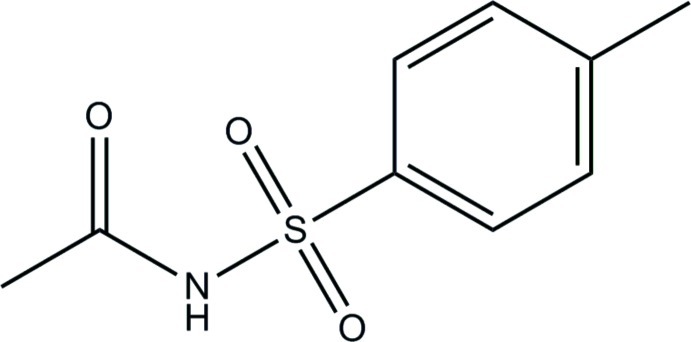


## Experimental

### 

#### Crystal data

C_9_H_11_NO_3_S
*M* *_r_* = 213.25
Monoclinic, 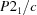

*a* = 9.2514 (6) Å
*b* = 5.1900 (3) Å
*c* = 20.5873 (13) Å
β = 95.070 (2)°
*V* = 984.63 (11) Å^3^
*Z* = 4
Mo *K*α radiation
μ = 0.31 mm^−1^
*T* = 100 K
0.27 × 0.19 × 0.08 mm

#### Data collection

Bruker APEX DUO CCD diffractometer
Absorption correction: multi-scan (*SADABS*; Bruker, 2009 ▶) *T*_min_ = 0.922, *T*_max_ = 0.976
15682 measured reflections
3114 independent reflections
2577 reflections with *I* > 2σ(*I*)
*R*_int_ = 0.038

#### Refinement

*R*[*F* ^2^ > 2σ(*F* ^2^)] = 0.032
*wR*(*F* ^2^) = 0.086
*S* = 1.07
3114 reflections
133 parameters
H atoms treated by a mixture of independent and constrained refinement
Δρ_max_ = 0.43 e Å^−3^
Δρ_min_ = −0.36 e Å^−3^

### 

Data collection: *APEX2* (Bruker, 2009 ▶); cell refinement: *SAINT* (Bruker, 2009 ▶); data reduction: *SAINT*; program(s) used to solve structure: *SHELXTL* (Sheldrick, 2008 ▶); program(s) used to refine structure: *SHELXTL*; molecular graphics: *SHELXTL*; software used to prepare material for publication: *SHELXTL* and *PLATON* (Spek, 2009 ▶).

## Supplementary Material

Crystal structure: contains datablock(s) global, I. DOI: 10.1107/S1600536812024658/hb6824sup1.cif

Structure factors: contains datablock(s) I. DOI: 10.1107/S1600536812024658/hb6824Isup2.hkl

Supplementary material file. DOI: 10.1107/S1600536812024658/hb6824Isup3.cml

Additional supplementary materials:  crystallographic information; 3D view; checkCIF report

## Figures and Tables

**Table 1:** Hydrogen-bond geometry (Å, °)

*D*—H⋯*A*	*D*—H	H⋯*A*	*D*⋯*A*	*D*—H⋯*A*
N1—H1*N*1⋯O1^i^	0.85 (2)	2.14 (2)	2.9586 (14)	161.2 (17)
C9—H9*A*⋯O3^ii^	0.98	2.49	3.4623 (15)	175
C9—H9*C*⋯O3^i^	0.98	2.32	3.2760 (14)	165

Symmetry codes: (i) 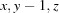; (ii) 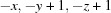.

**Fig. 1.**
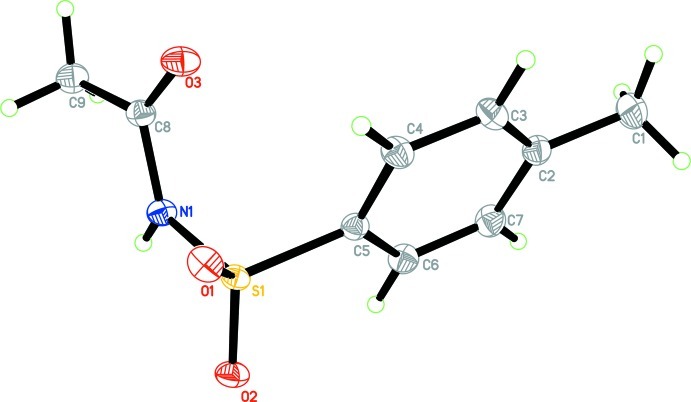
The molecular structure of the title compound with 50% probability displacement ellipsoids.

**Fig. 2.**
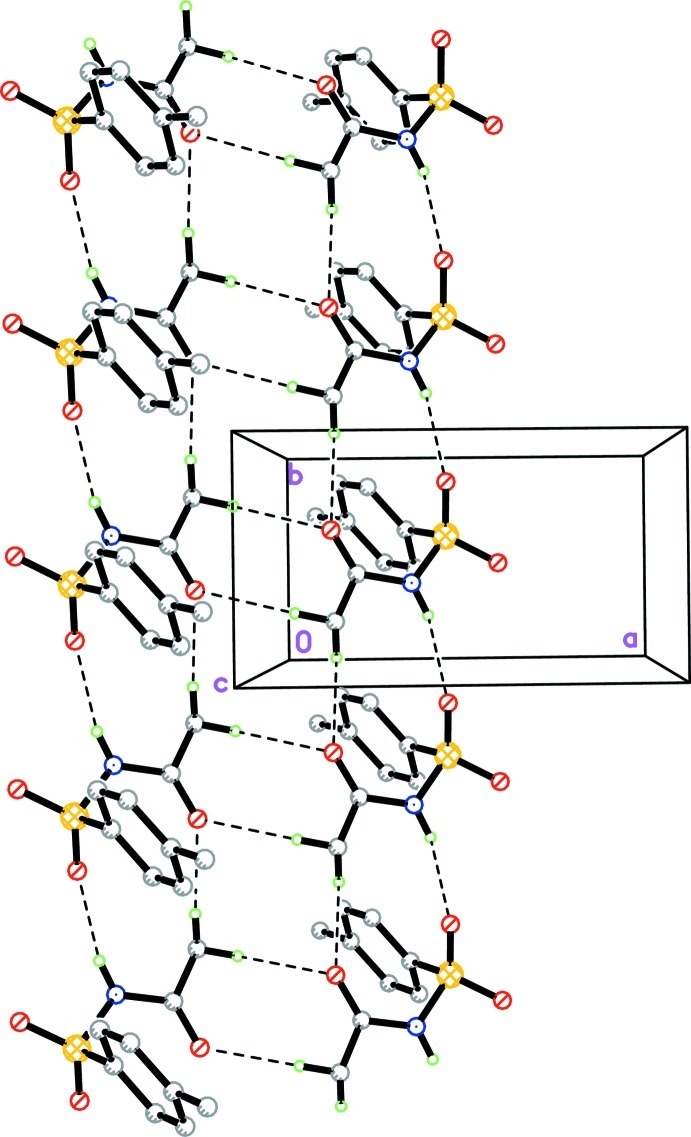
The crystal packing of the title compound. The dashed lines represent the hydrogen bonds. For clarity sake, hydrogen atoms not involved in hydrogen bonding have been omitted.

## supplementary crystallographic information

### Comment

Many of the compounds containing sulfonamide groups possess a broad spectrum of biological activities (Ashton *et al.*, 1994; Heidler & Link, 2005) and can be used as herbicides (Kamoshita *et al.*, 1987). In addition, the nature and position of substituents play a significant role on the crystal structures of *N*-(aryl)-amides and *N*-(aryl)-sulfonamides (Gowda *et al.*, 2007, 2010; Shakuntala *et al.*, 2011*a*,b; Henschel *et al.*, 1996). In view of the importance of the biological activities of sulfonamide containing compounds, we report herein the crystal structure of the title compound.

The asymmetric unit of the title compound is shown in Fig. 1. The C═O and N—H bonds in the amide group [C8/O3/N1/H1N1; maximum deviation = 0.0439 (57) Å at atom N1] are *trans* to each other, similar to that observed in related structures (Gowda *et al.*, 2010; Shakuntala *et al.*, 2011*a*,b). The mean plane of the benzene ring (C2–C7) forms a dihedral angle of 76.7 (3)° with the mean plane of amide group.

In the crystal (Fig.2), molecules are linked by a pair of C9—H9A···O3 hydrogen bonds (Table 1) into inversion dimers with an *R*_2_^2^(8) ring motif. The dimers are further connected by N1—H1N1···O1 and C9—H9C···O3 hydrogen bonds (Table 1) into an infinite tape along *b* axis.

### Experimental

To a vigorously stirred mixture of 4-methylbenzenesulphonamide and silica sulfuric acid, acid chloride or acid anhydride was added at RT. The progress of the reaction was monitored by TLC. After completion of the reaction, ethyl acetate was added and the solid catalyst was removed by filtration. The filtrate was washed with water, dried and evaporated. The crude product was purified by recrystallization from ethanolic solution to yield colourless plates of the title compound.

### Refinement

The atom H1N1 was located in a difference fourier map and refined freely [N1—H1N1 = 0.851 (19) Å]. The remaining H atoms were positioned geometrically [C—H = 0.95 and 0.98 Å] and refined using a riding model with *U*_iso_(H) = 1.2 or 1.5*U*_eq_(C). A rotating group model was applied to the methyl group.

### Crystal data

**Table:**

C_9_H_11_NO_3_S	*F*(000) = 448
*M**_r_* = 213.25	*D*_x_ = 1.439 Mg m^−^^3^
Monoclinic, *P*2_1_/*c*	Mo *K*α radiation, λ = 0.71073 Å
Hall symbol: -P 2ybc	Cell parameters from 5205 reflections
*a* = 9.2514 (6) Å	θ = 2.8–30.9°
*b* = 5.1900 (3) Å	µ = 0.31 mm^−^^1^
*c* = 20.5873 (13) Å	*T* = 100 K
β = 95.070 (2)°	Plate, colourless
*V* = 984.63 (11) Å^3^	0.27 × 0.19 × 0.08 mm
*Z* = 4	

### Data collection

**Table:**

Bruker APEX DUO CCD diffractometer	3114 independent reflections
Radiation source: fine-focus sealed tube	2577 reflections with *I* > 2σ(*I*)
Graphite monochromator	*R*_int_ = 0.038
φ and ω scans	θ_max_ = 31.0°, θ_min_ = 2.0°
Absorption correction: multi-scan (*SADABS*; Bruker, 2009)	*h* = −12→13
*T*_min_ = 0.922, *T*_max_ = 0.976	*k* = −7→7
15682 measured reflections	*l* = −29→29

### Refinement

**Table:**

Refinement on *F*^2^	Primary atom site location: structure-invariant direct methods
Least-squares matrix: full	Secondary atom site location: difference Fourier map
*R*[*F*^2^ > 2σ(*F*^2^)] = 0.032	Hydrogen site location: inferred from neighbouring sites
*wR*(*F*^2^) = 0.086	H atoms treated by a mixture of independent and constrained refinement
*S* = 1.07	*w* = 1/[σ^2^(*F*_o_^2^) + (0.0405*P*)^2^ + 0.3454*P*] where *P* = (*F*_o_^2^ + 2*F*_c_^2^)/3
3114 reflections	(Δ/σ)_max_ < 0.001
133 parameters	Δρ_max_ = 0.43 e Å^−^^3^
0 restraints	Δρ_min_ = −0.36 e Å^−^^3^

### Special details

**Table:**

Experimental. The crystal was placed in the cold stream of an Oxford Cryosystems Cobra open-flow nitrogen cryostat (Cosier & Glazer, 1986) operating at 100.0 (1) K.
Geometry. All e.s.d.'s (except the e.s.d. in the dihedral angle between two l.s. planes) are estimated using the full covariance matrix. The cell e.s.d.'s are taken into account individually in the estimation of e.s.d.'s in distances, angles and torsion angles; correlations between e.s.d.'s in cell parameters are only used when they are defined by crystal symmetry. An approximate (isotropic) treatment of cell e.s.d.'s is used for estimating e.s.d.'s involving l.s. planes.
Refinement. Refinement of *F*^2^ against ALL reflections. The weighted *R*-factor *wR* and goodness of fit *S* are based on *F*^2^, conventional *R*-factors *R* are based on *F*, with *F* set to zero for negative *F*^2^. The threshold expression of *F*^2^ > σ(*F*^2^) is used only for calculating *R*-factors(gt) *etc*. and is not relevant to the choice of reflections for refinement. *R*-factors based on *F*^2^ are statistically about twice as large as those based on *F*, and *R*- factors based on ALL data will be even larger.

### Fractional atomic coordinates and isotropic or equivalent isotropic displacement parameters (Å^2^)

**Table:**

	*x*	*y*	*z*	*U*_iso_*/*U*_eq_	
S1	0.45077 (3)	0.60012 (5)	0.397692 (13)	0.01332 (8)	
O1	0.45409 (9)	0.84344 (17)	0.43094 (4)	0.01789 (18)	
O2	0.58468 (9)	0.47512 (18)	0.38706 (4)	0.01848 (18)	
O3	0.16276 (10)	0.63022 (17)	0.44799 (5)	0.02044 (19)	
N1	0.36478 (11)	0.3887 (2)	0.44001 (5)	0.01466 (19)	
C1	0.09268 (15)	0.7079 (3)	0.13904 (6)	0.0264 (3)	
H1A	0.0199	0.8436	0.1421	0.040*	
H1B	0.0439	0.5443	0.1277	0.040*	
H1C	0.1566	0.7532	0.1053	0.040*	
C2	0.18124 (13)	0.6809 (3)	0.20370 (6)	0.0186 (2)	
C3	0.16453 (13)	0.8551 (2)	0.25401 (6)	0.0191 (2)	
H3A	0.0962	0.9912	0.2474	0.023*	
C4	0.24621 (13)	0.8331 (2)	0.31380 (6)	0.0180 (2)	
H4A	0.2341	0.9525	0.3479	0.022*	
C5	0.34592 (12)	0.6332 (2)	0.32277 (5)	0.0143 (2)	
C6	0.36550 (13)	0.4571 (2)	0.27322 (6)	0.0179 (2)	
H6A	0.4346	0.3221	0.2798	0.022*	
C7	0.28242 (14)	0.4823 (3)	0.21408 (6)	0.0202 (2)	
H7A	0.2946	0.3625	0.1801	0.024*	
C8	0.22879 (12)	0.4332 (2)	0.46235 (5)	0.0153 (2)	
C9	0.17645 (13)	0.2221 (2)	0.50349 (6)	0.0196 (2)	
H9A	0.0778	0.2610	0.5143	0.029*	
H9B	0.2409	0.2074	0.5437	0.029*	
H9C	0.1763	0.0591	0.4795	0.029*	
H1N1	0.4057 (19)	0.242 (4)	0.4444 (8)	0.029 (4)*	

### Atomic displacement parameters (Å^2^)

**Table:**

	*U*^11^	*U*^22^	*U*^33^	*U*^12^	*U*^13^	*U*^23^
S1	0.01197 (13)	0.01170 (13)	0.01619 (13)	0.00063 (9)	0.00059 (9)	−0.00001 (10)
O1	0.0193 (4)	0.0129 (4)	0.0210 (4)	−0.0007 (3)	−0.0014 (3)	−0.0024 (3)
O2	0.0130 (4)	0.0194 (4)	0.0232 (4)	0.0032 (3)	0.0022 (3)	0.0007 (3)
O3	0.0175 (4)	0.0155 (4)	0.0287 (5)	0.0033 (3)	0.0042 (3)	−0.0004 (3)
N1	0.0147 (4)	0.0111 (4)	0.0185 (4)	0.0021 (4)	0.0029 (3)	0.0017 (4)
C1	0.0237 (6)	0.0355 (8)	0.0192 (6)	−0.0010 (6)	−0.0032 (5)	0.0023 (5)
C2	0.0167 (5)	0.0220 (6)	0.0170 (5)	−0.0033 (5)	0.0009 (4)	0.0018 (4)
C3	0.0177 (5)	0.0182 (6)	0.0210 (5)	0.0029 (4)	−0.0010 (4)	0.0019 (4)
C4	0.0190 (5)	0.0153 (5)	0.0196 (5)	0.0031 (4)	0.0008 (4)	−0.0010 (4)
C5	0.0139 (5)	0.0138 (5)	0.0154 (5)	−0.0007 (4)	0.0018 (4)	0.0007 (4)
C6	0.0194 (5)	0.0156 (5)	0.0191 (5)	0.0025 (4)	0.0033 (4)	−0.0012 (4)
C7	0.0227 (6)	0.0210 (6)	0.0172 (5)	0.0006 (5)	0.0026 (4)	−0.0029 (4)
C8	0.0134 (5)	0.0159 (5)	0.0163 (5)	−0.0014 (4)	0.0004 (4)	−0.0035 (4)
C9	0.0180 (5)	0.0186 (6)	0.0222 (5)	−0.0032 (5)	0.0028 (4)	0.0005 (5)

### Geometric parameters (Å, º)

**Table:**

S1—O2	1.4323 (9)	C3—C4	1.3910 (16)
S1—O1	1.4354 (9)	C3—H3A	0.9500
S1—N1	1.6486 (10)	C4—C5	1.3899 (16)
S1—C5	1.7563 (12)	C4—H4A	0.9500
O3—C8	1.2141 (14)	C5—C6	1.3934 (16)
N1—C8	1.3964 (14)	C6—C7	1.3874 (17)
N1—H1N1	0.851 (19)	C6—H6A	0.9500
C1—C2	1.5066 (17)	C7—H7A	0.9500
C1—H1A	0.9800	C8—C9	1.4917 (17)
C1—H1B	0.9800	C9—H9A	0.9800
C1—H1C	0.9800	C9—H9B	0.9800
C2—C3	1.3936 (17)	C9—H9C	0.9800
C2—C7	1.3962 (18)		
			
O2—S1—O1	119.27 (5)	C5—C4—C3	118.76 (11)
O2—S1—N1	104.10 (5)	C5—C4—H4A	120.6
O1—S1—N1	108.93 (5)	C3—C4—H4A	120.6
O2—S1—C5	109.15 (5)	C4—C5—C6	121.33 (11)
O1—S1—C5	108.64 (5)	C4—C5—S1	119.88 (9)
N1—S1—C5	105.92 (5)	C6—C5—S1	118.78 (9)
C8—N1—S1	123.71 (9)	C7—C6—C5	118.88 (11)
C8—N1—H1N1	121.2 (12)	C7—C6—H6A	120.6
S1—N1—H1N1	114.9 (12)	C5—C6—H6A	120.6
C2—C1—H1A	109.5	C6—C7—C2	121.05 (11)
C2—C1—H1B	109.5	C6—C7—H7A	119.5
H1A—C1—H1B	109.5	C2—C7—H7A	119.5
C2—C1—H1C	109.5	O3—C8—N1	120.50 (11)
H1A—C1—H1C	109.5	O3—C8—C9	125.13 (11)
H1B—C1—H1C	109.5	N1—C8—C9	114.37 (10)
C3—C2—C7	118.82 (11)	C8—C9—H9A	109.5
C3—C2—C1	120.60 (12)	C8—C9—H9B	109.5
C7—C2—C1	120.57 (12)	H9A—C9—H9B	109.5
C4—C3—C2	121.15 (11)	C8—C9—H9C	109.5
C4—C3—H3A	119.4	H9A—C9—H9C	109.5
C2—C3—H3A	119.4	H9B—C9—H9C	109.5
			
O2—S1—N1—C8	178.75 (9)	O2—S1—C5—C6	27.10 (11)
O1—S1—N1—C8	50.50 (11)	O1—S1—C5—C6	158.66 (9)
C5—S1—N1—C8	−66.20 (10)	N1—S1—C5—C6	−84.45 (10)
C7—C2—C3—C4	−0.19 (19)	C4—C5—C6—C7	−0.52 (18)
C1—C2—C3—C4	−179.65 (12)	S1—C5—C6—C7	179.77 (9)
C2—C3—C4—C5	0.09 (19)	C5—C6—C7—C2	0.41 (19)
C3—C4—C5—C6	0.27 (18)	C3—C2—C7—C6	−0.06 (19)
C3—C4—C5—S1	179.99 (9)	C1—C2—C7—C6	179.40 (12)
O2—S1—C5—C4	−152.62 (10)	S1—N1—C8—O3	4.19 (16)
O1—S1—C5—C4	−21.06 (11)	S1—N1—C8—C9	−175.99 (8)
N1—S1—C5—C4	95.83 (10)		

### Hydrogen-bond geometry (Å, º)

**Table:**

*D*—H···*A*	*D*—H	H···*A*	*D*···*A*	*D*—H···*A*
N1—H1*N*1···O1^i^	0.85 (2)	2.14 (2)	2.9586 (14)	161.2 (17)
C9—H9*A*···O3^ii^	0.98	2.49	3.4623 (15)	175
C9—H9*C*···O3^i^	0.98	2.32	3.2760 (14)	165

Symmetry codes: (i) *x*, *y*−1, *z*; (ii) −*x*, −*y*+1, −*z*+1.

## References

[bb1] Ashton, W. T., Chang, L. L., Flanagan, K. L., Hutchins, S. M., Naylor, E. M., Chakravarty, P. K., Patchett, A. A., Greenlee, W. J., Chen, T. B., Faust, K. A., Chang, R. S. L., Lotti, V. J., Zingaro, G. J., Schorn, T. W., Siegl, P. K. S. & Kivlighn, S. D. (1994). *J. Med. Chem.* **37**, 2808–2824. 10.1021/jm00043a020 8064808

[bb2] Bruker (2009). *SADABS*, *APEX2* and *SAINT* Bruker AXS Inc., Madison, Wisconsin, USA.

[bb12] Cosier, J. & Glazer, A. M. (1986). *J. Appl. Cryst.* **19**, 105–107.

[bb3] Gowda, B. T., Foro, S. & Fuess, H. (2007). *Acta Cryst.* E**63**, o2597.

[bb4] Gowda, B. T., Foro, S., Nirmala, P. G. & Fuess, H. (2010). *Acta Cryst.* E**66**, o1284. 10.1107/S1600536810015849 PMC2979412 21579383

[bb5] Heidler, P. & Link, A. (2005). *Bioorg. Med. Chem.* **13**, 585–599. 10.1016/j.bmc.2004.10.045 15653327

[bb6] Henschel, D., Hiemisch, O., Blaschette, A. & Jones, P. G. (1996). *Z. Naturforsch. Teil B*, **51**, 1313–1315.

[bb7] Kamoshita, K., Matsumoto, H. & Nagano, E. (1987). US Patent No. 4 670 046.

[bb8] Shakuntala, K., Foro, S. & Gowda, B. T. (2011*a*). *Acta Cryst.* E**67**, o1097. 10.1107/S1600536811012785 PMC3089153 21754417

[bb9] Shakuntala, K., Foro, S. & Gowda, B. T. (2011*b*). *Acta Cryst.* E**67**, o1187. 10.1107/S1600536811014164 PMC3089174 21754489

[bb10] Sheldrick, G. M. (2008). *Acta Cryst.* A**64**, 112–122. 10.1107/S0108767307043930 18156677

[bb11] Spek, A. L. (2009). *Acta Cryst.* D**65**, 148–155. 10.1107/S090744490804362X PMC2631630 19171970

